# Interaction between Arsenic Exposure from Drinking Water and Genetic Polymorphisms on Cardiovascular Disease in Bangladesh: A Prospective Case-Cohort Study

**DOI:** 10.1289/ehp.1307883

**Published:** 2015-01-09

**Authors:** Fen Wu, Farzana Jasmine, Muhammad G. Kibriya, Mengling Liu, Xin Cheng, Faruque Parvez, Tariqul Islam, Alauddin Ahmed, Muhammad Rakibuz-Zaman, Jieying Jiang, Shantanu Roy, Rachelle Paul-Brutus, Vesna Slavkovich, Tariqul Islam, Diane Levy, Tyler J. VanderWeele, Brandon L. Pierce, Joseph H. Graziano, Habibul Ahsan, Yu Chen

**Affiliations:** 1 Department of Population Health, New York University School of Medicine, New York, New York, USA; 2 Department of Health Studies,; 3 Department of Medicine,; 4 Department of Human Genetics, and; 5 Comprehensive Cancer Center, The University of Chicago, Chicago, Illinois, USA; 6 Department of Environmental Health Sciences, Mailman School of Public Health, Columbia University, New York, New York, USA; 7 U-Chicago Research Bangladesh, Ltd., Dhaka, Bangladesh; 8 Department of Epidemiology, and; 9 Department of Biostatistics, Harvard School of Public Health, Boston, Massachusetts, USA

## Abstract

Background: Epidemiologic data on genetic susceptibility to cardiovascular effects of arsenic exposure from drinking water are limited.

Objective: We investigated whether the association between well-water arsenic and cardiovascular disease (CVD) differed by 170 single nucleotide polymorphisms (SNPs) in 17 genes related to arsenic metabolism, oxidative stress, inflammation, and endothelial dysfunction.

Method: We conducted a prospective case-cohort study nested in the Health Effects of Arsenic Longitudinal Study, with a random subcohort of 1,375 subjects and 447 incident fatal and nonfatal cases of CVD. Well-water arsenic was measured in 2000 at baseline. The CVD cases, 56 of which occurred in the subcohort, included 238 coronary heart disease cases, 165 stroke cases, and 44 deaths due to other CVD identified during follow-up from 2000 to 2012.

Results: Of the 170 SNPs tested, multiplicative interactions between well-water arsenic and two SNPs, rs281432 in *ICAM1* (*p*_adj_ = 0.0002) and rs3176867 in *VCAM1* (*p*_adj_ = 0.035), were significant for CVD after adjustment for multiple testing. Compared with those with GC or CC genotype in rs281432 and lower well-water arsenic, the adjusted hazard ratio (aHR) for CVD was 1.82 (95% CI: 1.31, 2.54) for a 1-SD increase in well-water arsenic combined with the GG genotype, which was greater than expected given aHRs of 1.08 and 0.96 for separate effects of arsenic and the genotype alone, respectively. Similarly, the joint aHR for arsenic and the rs3176867 CC genotype was 1.34 (95% CI: 0.95, 1.87), greater than expected given aHRs for their separate effects of 1.02 and 0.84, respectively.

Conclusions: Associations between CVD and arsenic exposure may be modified by genetic variants related to endothelial dysfunction.

Citation: Wu F, Jasmine F, Kibriya MG, Liu M, Cheng X, Parvez F, Islam T, Ahmed A, Rakibuz-Zaman M, Jiang J, Roy S, Paul-Brutus R, Slavkovich V, Islam T, Levy D, VanderWeele TJ, Pierce BL, Graziano JH, Ahsan H, Chen Y. 2015. Interaction between arsenic exposure from drinking water and genetic polymorphisms on cardiovascular disease in Bangladesh: a prospective case-cohort study. Environ Health Perspect 123:451–457; http://dx.doi.org/10.1289/ehp.1307883

## Introduction

Arsenic (As) exposure from drinking water has been associated with an increased risk of cardiovascular disease (CVD) (Moon et al. 2012). Some studies have indicated that genetic polymorphisms could modify the cardiovascular effects of As exposure (Hsieh et al. 2008, 2011; Hsueh et al. 2005; Wang et al. 2007; Wu et al. 2010). However, the available data have limitations such as small sample sizes and inclusion of one genetic variant in a few genes.

Arsenic in drinking water is present as As^V^ and As^III^, known as inorganic As (iAs). The metabolism of As involves alternating reduction and methylation in which As^V^ is first reduced to As^III^, followed by methylation to monomethylarsonic acid (MMA^V^) and MMA^III^, and then methylation to dimethylarsinic acid (DMA^V^) and DMA^III^. The enzymatic regulation of As metabolism is partially known, with evidence for a role of purine nucleoside phosphorylase (PNP), which reduces As^V^, and of glutathione *S*-transferase omega 1 (GSTO1), which reduces all the pentavalent As species. Other enzymes of the GST family [i.e., GST mu 1 (GSTM1), GST pi 1 (GSTP1), and GST theta 1 (GSTT1)] play a role in cellular antioxidant defense mechanisms by catalyzing the reduction of potentially harmful peroxides. Key enzymes involved in the one-carbon methylation of As with *S-*adenosyl methionine (SAM) as the methyl donor include arsenic-3-methyltransferase (AS3MT), methylenetetrahydrofolate reductase (MTHFR), cystathionine beta-synthase (CBS), and PNP. AS3MT methylates the trivalent As species; MTHFR catalyzes the conversion of 5,10-methylenetetrahydrofolate to 5-methyltetrahydrofolate and ultimately promotes the formation of SAM; and CBS is involved in the conversion of homocysteine to cystathionine, a substrate for glutathione synthesis. Genetic polymorphisms in *PNP*, *GSTO1*, *GSTM1*, *GSTP1*, *GSTT1*, *AS3MT*, *MTHFR*, and *CBS* have been associated with the distribution of As metabolites in urine (Agusa et al. 2010; Pierce et al. 2012; Porter et al. 2010; Steinmaus et al. 2007; Yu et al. 2003). Some polymorphisms in *MTHFR*, *CBS*, *GSTO1*, *GSTM1*, and *GSTT1* have been associated with CVD risk (Kelly et al. 2002; Klerk et al. 2002; Kolsch et al. 2004; Olshan et al. 2003; Pezzini et al. 2002; Roest et al. 2001; Wang et al. 2002). However, no studies have investigated whether these polymorphisms may modify CVD risk associated with As exposure.

Experimental studies have indicated that As exposure may lead to CVD through the promotion of oxidative stress, inflammation, and endothelial dysfunction. In humans, arsenic exposure has been associated with higher circulating levels of soluble intercellular adhesion molecule-1 (sICAM-1) and soluble vascular adhesion molecule-1 (sVCAM-1) (Chen et al. 2007; Karim et al. 2013; Wu et al. 2012). Thus, genes involved in oxidative stress, such as heme oxygenase 1 (*HMOX1*), nitric oxide synthase 3 (*NOS3*), superoxide dismutase 2 (*SOD2*), and alpha polypeptide (*CYBA*); genes involved in inflammation, such as apolipoprotein E (*APOE*), tumor necrosis factor (*TNF*), and interleukin 6 (*IL6*); and genes involved in endothelial dysfunction, such as *ICAM1*, *VCAM1*, and sphingosine-1-phosphate receptor 1 (*S1PR1*), may also modify cardiovascular effects of As exposure. Some genetic polymorphisms in *HMOX1*, *NOS3*, *SOD2*, *CYBA*, and *APOE* have been shown to modify As-induced cardiovascular outcomes (Hsieh et al. 2008; Hsueh et al. 2005; Wu et al. 2010).

We conducted a prospective case-cohort study to investigate whether the associations between As exposure and CVD risk are modified by genetic polymorphisms in 18 genes related to As metabolism, oxidative stress, inflammation, and endothelial dysfunction.

## Methods

*The parent Health Effects of Arsenic Longitudinal Study (HEALS)*. Details of HEALS have been described previously (Ahsan et al. 2006). Briefly, we recruited 11,746 married adults (original cohort) during 2000–2002 and an additional 8,287 participants (expansion cohort) in 2006–2008, with a participation rate of 97%. The cohort is actively followed every 2 years with in-person visits, and passively followed with interim health surveys at a field clinic established solely for the cohort participants. Informed consent was obtained from the study participants, and the study procedures were approved by the Ethical Committee of the Bangladesh Medical Research Council and the Institutional Review Boards of Columbia University and the University of Chicago.

*Selection of cases and subcohort*. Details of the case-cohort study design have been described elsewhere (Chen et al. 2013b). All participants in HEALS who provided urine samples and were free of a history of CVD at baseline were eligible for this study (*n* = 19,489). Cases were classified according to the earliest CVD event that occurred. CVD cases were those who were diagnosed with coronary heart disease (CHD) or stroke, or those with any CVD as the cause of death between baseline and 20 September 2012. Cases were coded according to the *International Classification of Diseases, 10th Revision* (ICD-10; codes I00–I99), which included fatal and nonfatal CHD (ICD-10 codes I20–I25), stroke (ICD-10 codes I60–I69), and other CVD (ICD-10 codes I05, I06, I08, I11, I27, I42, I46, I47, and I50). Our outcomes of interest included *a*) overall CVD, consisting of any CVD deaths and those with nonfatal CHD, stroke, or other CVD; *b*) fatal and nonfatal CHD; and *c*) fatal and nonfatal stroke. We randomly selected 1,902 subjects from all the eligible participants at baseline as the subcohort (see Supplemental Material, Figure S1). After excluding participants without blood samples, the final study population included 1,375 subcohort members and 447 incident cases, 56 of which occurred in the subcohort. Demographic and As exposure variables were similar comparing those who gave blood and the overall cohort (Chen et al. 2013b).

*Assessment of causes of death and incident cases*. Briefly, we adapted a validated verbal autopsy procedure to ascertain the causes of death in cohort participants (Chen et al. 2011a). During the follow-up, upon receipt of a death report from family or neighbors, a study physician and a trained social worker administered the verbal autopsy form to the next of kin. An outcome assessment committee, consisting of physicians and a consulting cardiologist and neurologist who were blinded to exposure status, reviewed these data monthly. Potential cases of non-fatal stroke and CHD and participants with heart disease symptoms were identified in active and passive follow-up and referred to trained physicians for further evaluation and diagnostic tests at the field clinic. All medical records of standard diagnostic tests were requested and reviewed by the outcome assessment committee. Nonfatal stroke and CHD were defined based on WHO criteria (Aho et al. 1980).

*Arsenic exposure measurements*. At baseline, prior to subject recruitment, water samples were collected from all 10,971 tube wells in the study area. Similar to our prior studies (Chen et al. 2011a, 2013a, 2013b, 2013c), we used the As concentration in the baseline well as the long-term As exposure level, rather than baseline urinary As, because baseline well-water As better measured long-term exposure in our study population. Total As concentration was analyzed by high-resolution inductively coupled plasma mass spectrometry (ICP-MS) with a detection limit of < 0.2 μg/L. Among the subcohort in the present study, 92% of the study participants used the index well as their exclusive source of drinking water at baseline. The average duration of index well use was 7.8 years prior to baseline, accounting for ≥ 20% of each participant’s lifetime. Spot urine samples were collected at baseline and all follow-up visits. Total urinary As concentration was measured by graphite furnace atomic absorption, using a PerkinElmer AAnalyst 600 graphite furnace system (PerkinElmer, Waltham, MA, USA) with a detection limit of 2 μg/L. Urinary creatinine level was analyzed using a method based on the Jaffé reaction (Slot 1965). We used urinary As assessed at follow-up visits to track the change in exposure during follow-up and calculated visit-to-visit change in urinary As by subtracting urinary As at later visit from urinary As at earlier visit, similar to our previous studies (Chen et al. 2011a, 2011b, 2013a, 2013b, 2013c). Urinary arsenic metabolites were measured in baseline urine samples as previously described (Chen et al. 2013b). This method employed high-performance liquid chromatography separation of arsenobetaine (AsB), arsenocholine (AsC), As^V^, As^III^, MMA, and DMA, followed by detection by ICP-MS with using a dynamic reaction cell. The detection limits were 0.2 μg/L for AsB and AsC, and 0.1 μg/L for all other metabolites. The long-term intraclass correlations for the percentage of MMA (MMA%) and DMA (DMA%) were all > 0.82 (Ahsan et al. 2007). MMA% was calculated by dividing the concentration of MMA by total urinary As excluding AsB and AsC.

*Selection of genes and single nucleotide polymorphisms (SNPs)*. Candidate genes were selected *a priori a*) if they are involved in As metabolism, *b*) if they have been shown to modify associations between CVD and As exposure in previous epidemiologic studies, and/or *c*) if As exposure has been associated with gene products (such as plasma sICAM-1 and sVCAM-1) identified as CVD predictors or risk factors in epidemiologic studies. We selected 18 candidate genes related to As metabolism (*PNP*, *GSTO1*, *GSTM1*, *GSTP1*, *GSTT1*, *AS3MT*, *MTHFR*, and *CBS*), oxidative stress (*HMOX1*, *NOS3*, *SOD2*, and *CYBA*), and inflammation/endothelial dysfunction (*APOE*, *TNF*, *IL6*, *ICAM1*, *VCAM1*, and *S1PR1*). We first selected tag SNPs from the International HapMap Project (HMP 2009) and SeattleSNPs Genome Variation Server (GVS 2013) using the *r*^2^-based Tagger program with a pairwise *r*^2^ > 0.8 and a minor allele frequency (MAF) ≥ 5%. The selection was performed separately for each ethnic group in the Hapmap/SeattleSNPs data to compile a list that included all the tag SNPs. We also selected validated, nonsynonymous SNPs with an MAF ≥ 5% from SeattleSNPs, and potentially functional SNPs from the F-SNP database (Lee and Shatkay 2008). In addition, we included SNPs that have been associated with CVD and/or phenotypic markers of interest in the literature.

*Genotyping and data cleaning*. A total of 384 SNPs with a good Illumina score (≥ 0.5) were genotyped using a GoldenGate assay (Illumina, San Diego, CA, USA); 28 SNPs were excluded due to assay failure. Concordance rates for all assays were > 99%. Of the 356 SNPs (see Supplemental Material, Table S1), we removed 186 SNPs because of poor genotyping efficiency (< 95%), monomorphic genotype data, deviation from Hardy–Weinberg equilibrium (< 0.0001), or low MAF (< 5%) in the subcohort, leaving 170 SNPs in 17 genes for analysis.

*Statistical analyses*. We computed person-years from baseline to the date of the first CVD event (any fatal CVD or nonfatal CHD, stroke, or other CVD, whichever occurred first), the date of death due to causes other than CVD event, or 20 September 2012, whichever occurred first. We tested interactions between As exposure and each of the SNPs of interest regardless of the significance of the main effects of As exposure or SNPs because this approach may capture important interactions limited to a small subset of subjects (Bermejo and Hemminki 2007; Le Marchand and Wilkens 2008). For each SNP we used the following Cox proportional hazards model:

*h*(*t*) = *h*_0_(*t*)exp[β_A_As + β_G_G + β_AG_As*G + β*_i_X_i_*], [1]

where *h*(*t*) is the expected hazard of CVD at time *t*, *h*_0_(*t*) is the baseline hazard of CVD, β_A_ is the coefficient associated with As exposure, β_G_ is the coefficient associated with genotype, As is baseline As exposure as a continuous variable, G represents the dichotomous SNP genotype, β_AG_ is the coefficient associated with gene–As interactions, As*G is the cross-product term for testing gene–As interactions, β_i_ is the coefficient associated with the *i* model covariates, and *X_i_* represents the *i* model covariates. As mentioned above, we considered baseline well-water As as the main exposure because it better measured long-term exposure in our study population. Because > 50% of the 170 SNPs have a variant genotype frequency < 5% and the dominant genetic model is often highly correlated with the additive genetic model (Lettre et al. 2007), we conducted all tests for gene–As interactions using dominant genetic model for better statistical power, similar to other gene–environment interaction studies (Garcia-Closas et al. 2013; Wu et al. 2011). SNPs were dichotomized under a dominant genetic model in which genotype with either one or two copies of a minor allele (variant allele) was combined; for SNPs with a negative β_AG_ indicating an antagonistic interaction, we used genotype without the minor allele as the “at-risk” genotype for easy interpretation. We conducted weighted analyses for case–cohort studies as described previously (Breslow et al. 2009) with person-time as the time scale. Standard errors were estimated using the robust variance estimator (Barlow 1994; Barlow et al. 1999). For each SNP, we estimated adjusted hazard ratios (aHRs) and their 95% confidence intervals (CIs) for any CVD, CHD, or stroke in association with *a*) a 1-SD increase in baseline well-water As among those without the at-risk genotype(s) (expβ_A_, an estimate of the independent effect of As); *b*) having the at-risk (versus reference) genotype(s) in the absence of As [expβ_G_, an estimate of the independent effect of the at-risk genotype(s)]; and *c*) a 1-SD increase in As among those with the at-risk genotype(s) [exp(β_A_ + β_G_ + β_AG_), an estimate of the joint effect of exposure to both As and the at-risk genotype(s)]. The significance of the multiplicative interaction was assessed by the *p*-value for the cross-product term between each SNP and As exposure (β_AG_). To account for multiple testing, we implemented false-discovery rate (FDR) correction (Benjamini and Hochberg 1995). Analyses were adjusted for CVD risk factors that might be related to As exposure, including sex, and baseline values of age (years), body mass index (BMI; kilograms per meter squared), educational attainment (years), smoking status (never, past, current), systolic blood pressure, and diabetes status (yes/no), as well as a time-varying covariate indicating visit-to-visit change in urinary As. Additional control for diastolic blood pressure did not materially change the results (data not shown). For the SNPs that remained significant, we also assessed interaction on the additive scale (synergy) by testing whether the estimated joint effect of As exposure and the at-risk SNP genotype(s) was greater than the sum of the independent effect estimates for As exposure and the SNP, respectively. We estimated relative excess risk for interaction (RERI) (Rothman et al. 1980) and its 95% CI using the standard delta method (Hosmer and Lemeshow 1992). The RERI is a measure of difference in excess relative risks, such that an RERI > 0 indicates synergy between the two risk factors, and a 95% CI that excludes zero corresponds to *p* < 0.05. For each SNP, we also estimated the proportion of the main effect of As exposure on CVD attributable to interaction as [RERI × *P*(G = 1)] ÷ [(HR_10_ ‒ 1) + (RERI × *P*(G = 1)], where *P*(G = 1) is the frequency of the at-risk genotype(s) in the subcohort and HR_10_ denotes the independent HR for a 1-SD increase in As exposure among those with the reference genotype(s) (VanderWeele and Tchetgen Tchetgen 2014). We also examined the joint effects of the significant SNPs with dichotomous well-water As, determined by the median value in the subcohort, on CVD. The test for multiplicative interaction was done using the cross-product term of the SNPs and well-water As, both expressed as dichotomous variables. We also used the SNAP (SNP Annotation and Proxy Search) web tool (Johnson et al. 2008; SNAP 2008) to identify untyped SNPs that are in strong linkage disequilibrium (LD) with the significant SNPs (*r*^2^ > 0.8).

Sensitivity analyses were conducted using an additive genetic model, with SNPs modeled as an ordinal variable coded as 0, 1, or 2 according to the number of variant alleles. If the multiplicative interaction between a SNP and well-water As was nominally significant (*p* < 0.05), we also tested the corresponding multiplicative interaction between the SNP and baseline urinary creatinine-adjusted As for the same outcome. Methylation efficiency measured using urinary arsenic metabolites such as urinary MMA%, which has been positively associated with CVD risk in our population (Chen et al. 2013b), may be a mediator through which genetic factors are related to CVD risk. Therefore, we did not adjust for urinary MMA% in the main analyses, but did conduct sensitivity analyses further adjusting for MMA% in the subpopulation with data on MMA% (*n* = 1,269). We ran separate models estimating the main effects of the 170 SNPs on CVD, CHD, and stroke, which were not adjusted for baseline well-water As and visit-to-visit change in urinary As. All other analyses were conducted using SAS, version 9.2 (SAS Institute Inc., Cary, NC, USA) and survival and survey packages in R, version 2.13.1 (R Core Team 2011).

## Results

A total of 447 cases of CVD were included, including 238 cases of CHD (93 fatal and 145 nonfatal), 165 cases of stroke (106 fatal and 59 nonfatal), and 44 deaths due to other heart diseases (Table 1). The subcohort was representative of the overall cohort in terms of demographics, lifestyle, and As exposure variables (see Supplemental Material, Table S2). Compared with the subcohort as a whole, which included 56 of the CVD cases, CVD cases were more likely to be men, older, and ever-smokers at baseline, and they were more likely to have diabetes, higher blood pressure, higher well-water As levels, and higher urinary MMA% at baseline (Table 1). Adjusted HRs for CVD, CHD, and stroke in association with a 1-SD increase in baseline well-water As were 1.21 (95% CI: 1.08, 1.37), 1.17 (95% CI: 1.01, 1.35), and 1.19 (95% CI: 1.02, 1.40), respectively (see Supplemental Material, Table S3).

**Table 1:** Baseline characteristics of the subcohort and incident cases of CVD, CHD, and stroke.*^a^*

Characteristic	CVD	CHD	Stroke	Subcohort^*b*^
Participants (*n*)	447	238	165	1,375
Male (%)	70.9	71.4	72.7	41.8
Age (years)	47.4 ± 9.7	45.4 ± 9.2	50.8 ± 8.9	38.6 ± 9.7
BMI (kg/m^2^)	20.4 ± 3.7	21.2 ± 4.0	19.5 ± 3.2	19.9 ± 3.3
Education level (years)	4.1 ± 4.3	4.8 ± 4.4	3.2 ± 3.9	3.1 ± 3.7
Cigarette smoking status (%)				
Ever-smokers, men	84.5	82.9	85.8	76.0
Ever-smokers, women	13.9	17.7	6.7	7.6
Systolic blood pressure (mmHg)	130.1 ± 26.6	128.2 ± 24.9	136.4 ± 28.9	116.5 ± 17.3
Diastolic blood pressure (mmHg)	81.8 ± 14.0	81.8 ± 13.2	84.1 ± 15.0	75.0 ± 10.9
Diabetes status (%)	8.5	7.1	10.1	2.3
Well-water As (μg/L)	97.9 ± 109.2	90.1 ± 99.8	99.5 ± 103.5	80.8 ± 101.3
Total urinary As (μg/g creatinine)	255.8 ± 236.6	243.5 ± 220.3	261.9 ± 247.6	257.6 ± 322.0
Urinary MMA%	14.6 ± 5.3	14.9 ± 5.5	14.6 ± 5.1	13.1 ± 5.0
Values are mean ± SD except where indicated.^***a***^Data on BMI, systolic blood pressure, diastolic blood pressure, diabetes status, well-water As, and total urinary As were missing for 8, 4, 4, 41, 16, and 9 subjects, respectively. Data on urinary MMA% were available for 1,269 subjects. Incident cases of CVD, CHD, and stroke included fatal and nonfatal cases. ^***b***^The subcohort included 56 CVD cases.				

The association between well-water As and overall CVD was significantly different according to genotypes of 24 SNPs in eight genes at the nominal level (Table 2). The multiplicative interaction between well-water As and 2 SNPs—rs281432 in *ICAM1* (*p*_adj_ = 0.0002) and rs3176867 in *VCAM1* (*p*_adj_ = 0.035)—remained significant after adjusting for multiple testing. For instance, the aHR for CVD was 1.82 (95% CI: 1.31, 2.54) for every 101.3-μg/L increase in well-water As combined with the rs281432 GG genotype, much greater than the multiplication of the independent aHRs for the GG genotype alone (0.96; 95% CI: 0.65, 1.42) and well-water As alone (1.08; 95% CI: 0.94, 1.25). The corresponding RERI was 0.78 (95% CI: 0.48, 1.08), indicating a synergy or positive interaction on the additive level. Similarly, the joint aHR for As and the rs3176867 CC genotype was 1.34 (95% CI: 0.95, 1.87), greater than the multiplication of the aHRs for their separate effects of 1.02 (95% CI: 0.85, 1.24) and 0.84 (95% CI: 0.58, 1.22), respectively, and the corresponding RERI was 0.47 (95% CI: 0.25, 0.69).The estimated proportion of the effect of a 1-SD increase in As exposure attributable to interaction was 70% (95% CI: 2.5, 138%) for *ICAM1* rs281432 and 91% (95% CI: 38, 143%) for *VCAM1* rs3176867 among those with the at-risk genotype.

**Table 2:** Nominally significant interactions between well-water arsenic and SNPs in CVD.

Gene	SNP	Genotype	MAF (%)	aHR (95% CI) well-water arsenic^*a *^	aHR (95% CI) SNP^*a *^	aHR (95% CI) joint^*a*^	*p*-Value^*b*^	*p*_*adj*_^*c*^
*APOE*	rs405509	AA vs. AC + CC	C (46.3)	1.10 (0.94, 1.29)	0.73 (0.49, 1.09)	1.09 (0.78, 1.53)	0.021	0.287
	rs7259620	GG vs. AG + AA	A (40.5)	1.17 (1.00, 1.37)	0.89 (0.60, 1.31)	1.35 (0.96, 1.89)	0.041	0.324
*AS3MT*	rs1046778	TC + CC vs. TT	C (34.8)	1.02 (0.82, 1.26)	0.92 (0.63, 1.33)	1.22 (0.87, 1.72)	0.038	0.324
	rs10748839	TC + CC vs. TT	C (43.5)	0.95 (0.71, 1.26)	1.03 (0.68, 1.56)	1.34 (0.91, 2.00)	0.046	0.324
	rs10786719	AG + GG vs. AA	G (43.8)	0.95 (0.71, 1.26)	0.99 (0.66, 1.50)	1.28 (0.87, 1.90)	0.046	0.324
	rs11191454	AG + GG vs. AA	G (17.2)	1.11 (0.96, 1.28)	0.83 (0.53, 1.32)	1.27 (0.87, 1.84)	0.016	0.287
	rs12573221	AC + CC vs. AA	C (12.1)	1.12 (0.97, 1.29)	0.76 (0.47, 1.24)	1.18 (0.81, 1.71)	0.040	0.324
	rs4290163	GT + TT vs. GG	T (42.2)	0.96 (0.73, 1.27)	0.94 (0.63, 1.41)	1.25 (0.85, 1.84)	0.036	0.324
	rs9527	GG vs. GA + AA	A (7.5)	0.95 (0.73, 1.24)	0.77 (0.47, 1.24)	1.00 (0.63, 1.61)	0.041	0.324
*CBS*	rs1005585	AG + GG vs. AA	G (7.8)	1.16 (1.02, 1.33)	0.54 (0.30, 0.96)	1.03 (0.67, 1.58)	0.006	0.274
	rs3788050	GT + TT vs. GG	T (8.2)	1.17 (1.02, 1.33)	0.61 (0.35, 1.06)	1.10 (0.73, 1.66)	0.015	0.287
	rs8132811	CT + TT vs. CC	T (13.0)	1.15 (1.00, 1.32)	0.59 (0.38, 0.92)	1.01 (0.71, 1.45)	0.002	0.141
*GSTO1*	rs1147611	CA + AA vs. CC	A (30.2)	1.05 (0.87, 1.27)	1.18 (0.80, 1.74)	1.65 (1.16, 2.36)	0.026	0.296
	rs11509438	GA + AA vs. GG	A (10.1)	1.18 (1.04, 1.35)	1.01 (0.64, 1.60)	1.70 (1.19, 2.41)	0.024	0.295
	rs2282326	AC + CC vs. AA	C (30.0)	1.07 (0.90, 1.29)	1.19 (0.82, 1.75)	1.66 (1.17, 2.36)	0.041	0.324
*ICAM1*	rs281432	GG vs. CG + CC	C (49.5)	1.08 (0.94, 1.25)	0.96 (0.65, 1.42)	1.82 (1.31, 2.54)	9.4 × 10^–7^	0.0002
*NOS3*	rs1800783	TA + AA vs. TT	A (22.4)	1.06 (0.89, 1.26)	0.76 (0.51, 1.12)	1.06 (0.75, 1.50)	0.022	0.287
	rs6951150	CT + TT vs. CC	T (22.2)	1.04 (0.87, 1.24)	0.73 (0.49, 1.08)	1.04 (0.73, 1.48)	0.012	0.287
*SOD2*	rs2758331	CA + AA vs. CC	C (48.2)	0.92 (0.67, 1.26)	0.77 (0.51, 1.18)	1.07 (0.72, 1.59)	0.021	0.287
	rs2758334	TC + CC vs. TT	T (48.2)	0.90 (0.69, 1.18)	0.70 (0.47, 1.05)	0.96 (0.66, 1.41)	0.009	0.287
	rs8031	TA + AA vs. TT	T (48.5)	0.93 (0.68, 1.27)	0.77 (0.51, 1.17)	1.02 (0.68, 1.51)	0.046	0.324
*VCAM1*	rs3176867	CC vs. TC + TT	T (28.7)	1.02 (0.85, 1.24)	0.84 (0.58, 1.22)	1.34 (0.95, 1.87)	0.0004	0.035
	rs3176871	GG vs. GA + AA	A (5.1)	0.85 (0.61, 1.19)	0.42 (0.24, 0.74)	0.55 (0.32, 0.95)	0.018	0.287
	rs3765685	AA vs. AG + GG	G (16.8)	0.95 (0.74, 1.23)	0.88 (0.59, 1.31)	1.20 (0.83, 1.74)	0.014	0.287
^***a***^Adjusted HR in association with a 1-SD increase in well-water arsenic (101.3 μg/L) and “at-risk” genotype(s) of SNPs, and joint effect between well-water arsenic and SNPs, adjusting for sex, age, BMI, smoking status (never, past, and current), educational attainment, systolic blood pressure, diabetes status, and change in creatinine-adjusted urinary As between visits. SNPs were dichotomized assuming dominant effects; for SNPs with a negative effect estimate for the interaction term, genotype without the minor allele was used as the “at-risk” genotype for easy interpretation and compared with the combined genotypes with one or two copies of the minor allele. ^***b***^Nominal *p*-values from 1 degree of freedom tests for multiplicative interactions between a 1-SD increase well-water arsenic and SNPs. ^***c***^FDR-adjusted *p*-values.								

Sensitivity analyses using an additive genetic model revealed similar interaction patterns (data not shown). The joint effects for the *ICAM1* rs281432 and *VCAM1* rs3176867 SNPs considered with dichotomous well-water As are presented in Table 3. The association between CVD and well-water As ≥ 45 μg/L was more pronounced among individuals with the at-risk genotypes. The aHR for CVD in association with both higher As exposure and the GG genotype of *ICAM1* rs281432 was 2.98 (95% CI: 1.87, 4.77), much greater than the independent aHRs for higher well-water As alone (1.67; 95% CI: 1.14, 2.43) or the GG genotype alone (1.35; 95% CI: 0.84, 2.18), with an RERI of 0.97 (95% CI: –0.31, 2.24). Similarly, the joint aHR for higher well-water As and the CC genotype of *VCAM1* rs3176867 was 2.13 (95% CI: 1.37, 3.31), compared with the aHRs for their separate effects of 1.30 (95% CI: 0.83, 2.05) and 0.89 (95% CI: 0.56, 1.41), respectively, with an RERI of 0.94 (95% CI: 0.22, 1.66). In the subset with data on urinary MMA%, effect estimates adjusting for urinary MMA% were similar (data not shown). The LD between selected SNPs within *ICAM1* and *VCAM1* was evaluated using Haploview software (Barrett et al. 2005). As shown in Supplemental Material, Figures S2 and S3, block structure, defined for SNP pairs showing a D prime of > 0.8, was not observed for both *ICAM1* rs281432 and *VCAM1* rs3176867 with other SNPs in the genes.

**Table 3:** Joint effect between selected SNPs and well-water arsenic on CVD.

SNP	Well-water arsenic^*a*^	Cases/subcohort (*n*)	HR (95% CI)^*b*^	*p* for interaction^*c*^	RERI (95% CI)
*ICAM1* (rs281432)
CG + CC	< 45	143/500	Reference	0.40	0.97 (–0.31, 2.24)
GG	< 45	43/173	1.35 (0.84, 2.18)
CG + CC	≥ 45	185/520	1.67 (1.14, 2.43)
GG	≥ 45	73/151	2.98 (1.87, 4.77)
*VCAM1* (rs3176867)
TC + TT	< 45	166/594	Reference	0.04	0.94 (0.22, 1.66)
CC	< 45	16/65	0.89 (0.56, 1.41)
TC + TT	≥ 45	222/609	1.30 (0.83, 2.05)
CC	≥ 45	27/42	2.13 (1.37, 3.31)
^***a***^Cut points for well-water arsenic were determined by the median value in the subcohort. ^***b***^Adjusted for sex, age, BMI, smoking status (never, past, current), educational attainment, systolic blood pressure, diabetes status, and change in creatinine-adjusted urinary arsenic between visits. ^***c***^Significance of interaction at the multiplicative scale.					

A total of 26 SNPs in nine genes showed nominally significant interactions with well-water As in CHD (see Supplemental Material, Table S4); however, none of them was significant after adjusting for multiple testing. The association between well-water As and stroke was significantly modified by 11 SNPs in six genes (see Supplemental Material, Table S4), and *ICAM1* rs281432 remained significant after FDR adjustment (*p*_adj_ = 0.014). The joint aHR for a 1-SD increase in well-water As and the rs281432 GG genotype was 1.85 (95% CI: 1.14, 3.01), much greater than the independent aHRs for well-water As alone (1.08; 95% CI: 0.90, 1.31) or the GG genotype alone (0.92; 95% CI: 0.52, 1.61), with an RERI of 0.84 (95% CI: 0.39, 1.30).

Many of the interactions between SNPs and well-water As were replicated when urinary As was used as the exposure variable (see Supplemental Material, Table S5). For instance, there was evidence of a synergistic interaction between *ICAM1* rs281432 and urinary As on CVD (*p* = 0.014) and stroke (*p* = 0.005). The aHR for CVD was 1.68 (95% CI: 1.12, 2.52) for a 1-SD increase (322 μg/g creatinine) in urinary As and the GG genotype, compared with the aHR for urinary As alone (1.01; 95% CI: 0.75, 1.35) or the GG genotype alone (1.16; 95% CI: 0.79, 1.72), with an RERI of 0.51 (95% CI: 0.14, 0.87). The joint aHR for stroke in association with As and the rs281432 GG genotype was 1.60 (95% CI: 0.87, 2.93), compared with the aHRs for their separate effects of 0.96 (95% CI: 0.67, 1.39) and 0.97 (95% CI: 0.55, 1.72), respectively, with an RERI of 0.66 (95% CI: 0.16, 1.17).

We also estimated the main effects of SNPs on CVD, CHD, and stroke (see Supplemental Material, Table S6). The *NOS3* rs2853792 AG/GG genotype and the *SOD2* rs5746088 GA/AA genotype were negatively associated with CVD and CHD after FDR adjustment. Carriers of at least one T allele in *MTHFR* rs1801133 were 2.33 times as likely to have stroke (95% CI: 1.51, 3.61) as those with the CC genotype. Estimates of the main effects of SNPs on CVD, CHD, and stroke were similar under the additive genetic model (data not shown).

## Discussion

We observed a significant interaction of well-water As with *ICAM1* rs128432 and *VCAM1* rs3176867 for CVD on both multiplicative and additive scales. The joint effect of susceptible genotypes and well-water As was greater than the sum of their single effects alone, with RERIs of 0.78 (95% CI: 0.48, 1.08) for rs128432 and 0.47 (95% CI: 0.25, 0.69) for rs3176867. As estimated, > 70% of the main effect of a 1-SD increase in As exposure among individuals carrying the at-risk genotype was attributable to synergism between these two exposures, stressing the importance of genetic susceptibility in CVD risk related to As exposure.

The findings on the main effects of well-water As in the present study confirm the findings of previous studies of the same population (Chen et al. 2011a, 2013b). With a larger sample size, we also estimated a positive association between well-water As and stroke, a finding consistent with some (Chiou et al. 1997; Meliker et al. 2007; Moon et al. 2013) but not all (Chen et al. 2011a, 2013b; Medrano et al. 2010; Wu et al. 1989) previous studies.

We observed significant interactions of well-water As with rs281432 in *ICAM1* and rs3176867 in *VCAM1* for CVD. The two genes encode cell adhesion molecules (CAMs), namely ICAM-1 and VCAM-1, that are expressed on the surface of activated endothelial cells in response to inflammatory stimuli and mediate the attachment of circulating leukocytes to the endothelium, an early step of atherosclerosis. Circulating levels of sICAM-1 and sVCAM-1 have been predictive of CVD risk in cohort studies (Blankenberg et al. 2001; Ridker et al. 1998). A significant positive association between As exposure and plasma levels of sICAM-1 and sVCAM-1 has been reported in several studies (Chen et al. 2007; Karim et al. 2013; Wu et al. 2012). Findings of the present study support a role of endothelial dysfunction in the underlying mechanisms of the cardiovascular effects of As exposure. Specifically, *ICAM1* rs281432 is an intronic SNP that has been studied in diabetes and subclinical atherosclerosis, although findings have been mixed (Bielinski et al. 2008; Ma et al. 2006; Yang et al. 2014). For instance, in the Multi-Ethnic Study of Atherosclerosis, Bielinski et al. (2008) observed no association between rs281432 and subclinical atherosclerosis. In Swedish Caucasians, the C allele of rs281432 was significantly associated with type 1 diabetes (Ma et al. 2006). In a recent study in a Chinese population, Yang et al. (2014) found a significantly higher frequency of a three-allele haplotype containing the rs281432 G allele in coronary atherosclerosis cases, and the G allele was associated with a significantly higher level of triglycerides. Bielinski et al. (2008, 2011) reported that, in Chinese and African Americans, individuals carrying the rs281432 GG genotype had higher circulating levels of sICAM-1 compared with those with the CC genotype. (Bielinski et al. 2008, 2011). It is thus plausible that individuals carrying the GG genotype of rs281432, who were genetically predisposed to endothelial dysfunction in response to inflammatory stimuli, were more affected by the cardiovascular effects of As exposure. *VCAM1* rs3176867 lies in the intron 4 and was not in LD with other SNPs in the gene (see Supplemental Material, Figure S3). We did not identify any prior reports linking rs3176867 to any biochemical or disease phenotypes, and therefore a biological basis for an interaction with well-water As could not be identified.

Other SNPs in genes involved in inflammation (*APOE* and *IL6*), oxidative stress (*NOS3* and *SOD2*), and As metabolism (*AS3MT*, *CBS*, *GSTO1*, and *MTHFR*) showed nominally significant interactions with well-water As for associations with CVD, CHD, or stroke. Several SNPs in *AS3MT* have been consistently associated with As metabolism across diverse populations (Engström et al. 2011; Pierce et al. 2012). However, none of the interactions between As exposure and SNPs in *AS3MT* was significant in the present study population after adjusting for multiple comparisons. Our findings suggest that disease-specific susceptibility may play a more critical role than susceptibility due to As metabolism in the association between As exposure and CVD risk.

Among the SNPs that were tested, *NOS3* rs2853792, *SOD2* rs5746088, and *MTHFR* rs1801133 were significantly associated with CVD in this population. *MTHFR* rs1801133 is a C > T missense variation, leading to reduced activity of the MTHFR enzyme (Frosst et al. 1995), elevated homocysteine levels, and lower folate levels (Gudnason et al. 1998). Epidemiologic studies have reported an association between the rs1801133 TT genotype and stroke risk (Kelly et al. 2002). Our data suggest that, consistent with other populations, individuals carrying the TT genotype of rs1801133 may be more susceptible to stroke in the Bangladeshi population. The literature on *NOS3* rs2853792 and *SOD2* rs5746088 is limited, and our findings require future investigation.

Strengths of this study include detailed data on As exposure at the individual level using well-water and urine samples with repeated measurements, the use of comprehensive genomic technologies to measure tag SNPs and known/putative functional SNPs, and inclusion of an ethnically homogeneous population, which reduces population stratification bias. Our study also has several limitations. First, our analyses focused on *a priori* selected SNPs in candidate genes. We therefore cannot exclude the role of other SNPs and other genes. The identified SNPs may be markers of the underlying causal variants, and their effects could be underestimated if LD is incomplete (Zondervan and Cardon 2004). Among the GIH population (Gujarati Indians in Houston, Texas) in the Hapmap 3 SNP data set, *ICAM1* rs281432 was not found, and no proxy SNPs were identified for *VCAM1* rs3176867 (SNAP; Johnson et al. 2008). Alternatively, *ICAM1* rs281432 and *VCAM1* rs3176867, which are both intronic SNPs, may perform a functional role by exerting a direct effect on gene splicing (Wang and Cooper 2007) and expression of noncoding RNAs (Ragvin et al. 2010), and subsequently interfere with structure and function of protein. Second, although we corrected for multiple testing using the FDR approach, further replication studies and mechanistic studies are needed. Finally, we did not have data on lipid profiles. Although previous studies did not suggest a significant role of As in lipid profiles (Wu et al. 2014), we cannot rule out potential confounding of lipid profiles or other unmeasured confounders.

## Conclusion

We estimated significant interactions of As exposure with *ICAM1* rs281432 and *VCAM1* rs3176867 on CVD. Our findings support the notion that genetic variants by themselves may not substantially impact disease risk, but in concert with environmental exposures, they may increase the risk of disease.

## Supplemental Material

(925 KB) PDF
