# Supplementary material for: Interaction between Arsenic Exposure from Drinking Water and Genetic Polymorphisms on Cardiovascular Disease in Bangladesh: A Prospective Case-Cohort Study
Source: Environ Health Perspect. 2015 Jan 9;123(5):451–7. doi: 10.1289/ehp.1307883 (PMC4421763; doi:10.1289/ehp.1307883)
Supplement: (925 KB) PDF [file ehp.1307883.s001.508.pdf]

Environmental Health Perspectives doi:10.1289/ehp.1307883

Supplemental Material

Interaction between Arsenic Exposure from Drinking Water and Genetic Polymorphisms on Cardiovascular Disease in Bangladesh: A Prospective Case-Cohort Study

Fen Wu, Farzana Jasmine, Muhammad G. Kibriya, Mengling Liu, Xin Cheng, Faruque Parvez, Tariqul Islam, Alauddin Ahmed, Muhammad Rakibuz-Zaman, Jieying Jiang, Shantanu Roy, Rachelle Paul-Brutus, Vesna Slavkovich, Tariqul Islam, Diane Levy, Tyler J. VanderWeele, Brandon L. Pierce, Joseph H. Graziano, Habibul Ahsan, and Yu Chen

Table S1. Detailed information on the selected SNPs.

db SNP ID[a]	SNP position	Alleles	Role [amino acid change]	Prior studies min MAF[b]	Prior studies max MAF[b]	Present study minor allele	Present study MAF
***APOE* 19q13.32**							
rs1081101	45408077	C/T	Promoter	-	-	T	0.000
rs12982192	45411259	C/T	Intron (boundary)	-	-	C	0.000
**rs405509**	45408836	A/C	Promoter	0.204	0.487	C	0.463
**rs429358**	45411941	C/T	Coding exon [C130R]	0.000	0.018	C	0.103
rs434132	45407720	C/G	Promoter	-	-	C	0.001
rs435380	45407118	C/T	Promoter	-	-	T	0.008
rs446037	45407437	A/C	Promoter	-	-	A	0.000
rs7256173	45407655	C/T	Promoter	-	-	T	0.020
**rs7259620**	45407788	A/G	Promoter	-	-	A	0.405
rs769445	45408312	C/T	Promoter	-	-	T	0.002
rs769451	45410911	G/T	Intron	0.007	0.014	G	0.001
rs769452	45411110	C/T	Coding exon [L46P]	-	-	C	0.000
rs877973	45409283	G/T	Intron	-	-	T	0.000
***AS3MT* 10q24.32**							
**rs1046778**	104661484	C/T	3' UTR	0.213	0.488	C	0.348
rs10509760	104634107	C/T	Intron	0.007	0.160	C	0.043
rs10509761	104632769	A/G	Intron (boundary)	0.000	0.175	G	0.000
**rs10748835**	104660256	A/G	Intron (boundary)	0.306	0.494	A	0.439
**rs10748839**	104953547	C/T	Promoter	0.324	0.488	C	0.435
**rs10786719**	104637992	A/G	Intron	0.306	0.494	G	0.438
**rs10883790**	104640955	A/C	Intron	0.121	0.304	C	0.250
**rs10883795**	104654577	C/T	Intron	0.191	0.432	C	0.348
**rs11191438**	104637864	C/G	Intron	0.306	0.494	C	0.436
**rs11191439**	104638723	C/T	Coding exon	0.007	0.149	C	0.054
**rs11191442**	104643596	A/T	Intron	0.146	0.273	A	0.251
**rs11191454**	104660004	A/G	Intron	0.000	0.305	G	0.172
**rs11191527**	104795134	C/T	Intron	0.061	0.189	T	0.158
rs11191659	105101701	C/T	Intron	0.005	0.169	T	0.044
**rs12573221**	104849144	A/C	3' UTR	0.012	0.108	C	0.121
**rs12774047**	104639738	A/G	Intron	0.048	0.163	A	0.156
rs17115203	104639969	C/T	Intron	0.000	0.093	C	0.002
**rs3740390**	104638480	A/G	Intron	0.019	0.293	A	0.175
**rs3740392**	104636855	A/G	Intron (boundary)	0.145	0.331	G	0.251
**rs3740393**	104636655	C/G	Intron (boundary)	0.099	0.293	C	0.189
rs3740394	104634474	C/T	Intron (boundary)	0.007	0.160	C	0.044
**rs4290163**	104610926	G/T	Promoter	0.255	0.488	T	0.422
**rs4917996**	104925829	A/C	Intron	0.324	0.494	C	0.447
**rs4919690**	104616500	C/T	Intron	0.078	0.292	C	0.110
**rs4919694**	104698978	C/T	Intron	0.053	0.149	C	0.090
**rs7085854**	104650251	C/T	Intron (boundary)	0.067	0.234	C	0.175
**rs7096169**	104618695	A/G	Intron	0.292	0.476	G	0.292
**rs7100709**	104649729	A/C	Intron	0.191	0.432	C	0.347
rs7907785	104641035	A/G	Intron	0.000	0.139	G	0.000
**rs9527**	104623578	A/G	3' UTR	0.073	0.292	A	0.075
***CBS* 21q22.3**							
**rs1005585**	44477033	A/G	Intron (boundary)	-	-	G	0.078
**rs1051319**	44473867	C/G	3' UTR	0.068	0.389	G	0.111
**rs11203172**	44480115	G/T	Intron	0.000	0.185	T	0.053
**rs11700748**	44473062	C/T	Downstream	0.136	0.500	T	0.389
rs11701048	44491425	C/T	Intron	0.000	0.208	T	0.048
rs11910385	44491109	G/T	Intron	-	-	G	0.041
rs12613	44473691	A/G	3' UTR	-	-	A	0.035
rs13046443	44477738	C/T	Intron	-	-	T	0.000
**rs1789953**	44482936	C/T	Intron	0.043	0.277	T	0.238
rs2014564	44481169	A/G	Intron	-	-	A	0.478
**rs2124459**	44475714	C/T	Intron	0.223	0.464	C	0.411
rs2124461	44475796	C/T	Intron	-	-	T	0.474
rs2298760	44486211	A/C	Intron	-	-	A	0.000
**rs234701**	44476759	A/G	Intron	-	-	A	0.093

db SNP ID[a]	SNP position	Alleles	Role [amino acid change]	Prior studies min MAF[b]	Prior studies max MAF[b]	Present study minor allele	Present study MAF
rs234704	44480283	A/G	Intron	-	-	A	0.206
**rs234705**	44483772	C/T	Intron	0.022	0.436	T	0.219
**rs234706**	44485350	A/G	Coding exon [Y233Y]	0.014	0.351	A	0.209
**rs234709**	44486964	C/T	Intron	0.435	0.435	T	0.307
**rs234713**	44487891	A/G	Intron	-	-	A	0.203
**rs234715**	44488395	G/T	Intron	0.023	0.236	T	0.165
**rs2849727**	44483453	C/T	Intron	-	-	T	0.339
**rs2851391**	44487404	C/T	Intron	0.236	0.473	T	0.477
**rs2851392**	44489977	G/T	Intron	-	-	T	0.345
rs34040148	44488631	A/C	Coding exon [K102Q]	-	-	C	0.000
**rs3788050**	44474663	G/T	Intron	0.036	0.309	T	0.082
rs3788053	44484723	A/G	Intron	0.068	0.116	G	0.023
rs397589	44490214	G/T	Intron	0.114	0.119	G	0.021
**rs4920037**	44481891	A/G	Intron	0.014	0.225	A	0.175
**rs6586281**	44478393	A/G	Intron (boundary)	-	-	A	0.078
**rs6586282**	44478497	C/T	Intron	0.000	0.255	T	0.077
rs6586283	44478680	C/T	Intron	0.318	0.318	C	0.005
**rs706208**	44473446	C/T	3' UTR	0.134	0.500	C	0.385
rs7276016	44489664	A/G	Intron	-	-	A	0.035
rs760124	44475953	A/G	Intron	-	-	G	0.012
rs8131163	44488031	C/T	Intron	-	-	T	0.000
**rs8132811**	44475877	C/T	Intron	0.080	0.373	T	0.130
rs9978863	44483233	A/G	Intron (boundary)	-	-	A	0.000
rs9982015	44490092	C/T	Intron	0.000	0.164	C	0.037
rs9983620	44493201	A/G	Intron	-	-	G	0.000
***CYBA* 16q24.3**							
**rs12709102**	88712319	A/G	Intron	0.084	0.464	G	0.316
rs12933505	88712767	A/G	Intron	0.092	0.494	G	0.000
rs13306295	88712618	C/T	Intron (boundary)	-	-	T	0.000
**rs13306296**	88717957	C/T	Promoter	-	-	T	0.098
rs16966653	88710636	C/G	Intron	-	-	G	0.000
rs3180279	88710833	C/G	Intron	0.167	0.454	G	0.384
rs3199601	88710095	C/T	Intron	-	-	T	0.042
**rs3794624**	88717074	A/G	Intron	0.108	0.405	A	0.107
**rs4782391**	88711466	A/G	Intron	-	-	A	0.076
rs4782393	88713501	A/G	Intron (boundary)	-	-	G	0.437
**rs9925947**	88709343	A/G	Downstream	-	-	A	0.318
rs9940427	88711819	G/T	Intron	0.000	0.032	T	0.004
***GSTM1* 1p13.3**							
**rs1634252**	110228690	A/G	Promoter	-	-	A	0.396
rs2071487	110233081	C/T	Intron (boundary)	0.291	0.307	T	0.427
rs2239892	110234286	C/T	Intron	0.039	0.139	C	0.147
rs412543	110229944	C/G	Promoter	0.054	0.218	C	0.039
rs4147563	110230103	C/T	Promoter	-	-	T	0.323
rs4147567	110232524	A/G	Intron	0.000	0.134	G	0.133
rs737497	110231592	A/G	Intron (boundary)	-	-	G	0.287
***GSTO1* 10q25.1**							
**rs1147611**	106025258	A/C	Intron	0.121	0.435	A	0.302
**rs11509438**	106027059	A/G	Coding exon [E208K]	0.006	0.096	A	0.101
**rs17116736**	106023387	A/G	Intron	-	-	G	0.012
rs2282326	106020398	A/C	Intron	0.187	0.435	C	0.300
**rs4925**	106022789	A/C	Coding exon [A140D]	0.042	0.383	A	0.168
***GSTP1* 11q13.2**							
rs1138272	67353579	C/T	Coding exon	0.000	0.096	T	0.048
**rs1695**	67352689	A/G	Coding exon	0.098	0.494	G	0.244
**rs4147581**	67351585	C/G	Intron (boundary)	0.065	0.472	C	0.324
rs5031031	67354104	A/G	3' UTR	-	-	G	0.000
**rs6591256**	67349899	A/G	Promoter	0.098	0.465	G	0.258
**rs749174**	67353253	C/T	Intron	0.079	0.451	T	0.213
rs8191431	67350054	C/T	Promoter	0.000	0.213	T	0.000
rs8191439	67351297	A/G	5' UTR	0.004	0.362	A	0.000
rs8191446	67351827	C/G	Intron	0.000	0.213	C	0.000

db SNP ID[a]	SNP position	Alleles	Role [amino acid change]	Prior studies min MAF[b]	Prior studies max MAF[b]	Present study minor allele	Present study MAF
rs8191449	67352381	A/G	Intron	-	-	A	0.000
**rs947895**	67354406	A/C	Downstream	0.079	0.458	A	0.212
***GSTT1* 22q11.23**							
rs2234953	24376833	A/G	Downstream	-	-	A	0.001
rs2266635	24384171	A/G	Coding exon [A21T]	0.005	0.015	A	0.012
rs4630	24376322	C/T	Downstream	-	-	C	0.067
***HMOX1* 22q12.3**							
rs11555832	35789869	C/T	3' UTR	0.011	0.431	C	0.000
**rs11912889**	35783617	A/G	Intron	0.029	0.476	A	0.087
**rs16995662**	35784998	A/G	Intron	-	-	G	0.080
rs17879606	35782659	C/T	Intron (boundary)	-	-	T	0.007
rs17880294	35783286	C/T	Intron	-	-	T	0.000
rs17882597	35786008	I/D[c]	Intron	-	-	D	0.000
rs17885185	35783776	C/T	Intron	-	-	T	0.000
**rs17885522**	35777856	A/C	Intron	-	-	A	0.095
rs17885925	35779223	C/T	Intron (boundary)	-	-	C	0.006
rs17886055	35787065	C/T	Intron	-	-	C	0.007
rs2003038	35788909	C/T	Intron	-	-	T	0.000
**rs2071748**	35777618	A/G	Intron	0.277	0.494	G	0.464
**rs2071749**	35783413	A/G	Intron	0.009	0.469	A	0.298
**rs2269534**	35786226	C/T	Intron	-	-	C	0.433
**rs2285112**	35789263	A/G	Intron	0.223	0.494	A	0.466
**rs4820192**	35777984	A/C	Intron	-	-	C	0.441
rs5755713	35782989	C/G	Coding exon [Q152H]	-	-	C	0.000
rs5755718	35786722	C/T	Intron	-	-	C	0.173
**rs5755720**	35786873	A/G	Intron	0.130	0.493	G	0.433
**rs5995098**	35787167	C/G	Intron	0.073	0.419	G	0.432
**rs5995099**	35787235	C/T	Intron	-	-	T	0.433
**rs6518952**	35782513	C/T	Intron	0.000	0.491	T	0.080
**rs8139532**	35779568	A/G	Intron	0.024	0.407	A	0.085
rs8140370	35779635	G/T	Intron	0.000	0.241	G	0.000
**rs8140669**	35779844	A/T	Intron	0.000	0.435	A	0.080
rs9607267	35781207	C/T	Intron	0.306	0.438	C	0.471
**rs9622194**	35786963	A/G	Intron	-	-	A	0.080
***ICAM1* 19p13.2**							
**rs281432**	10390658	C/G	Intron	0.250	0.482	C	0.495
rs281433	10392223	A/C	Intron	0.000	0.127	C	0.002
**rs3093032**	10396336	C/T	Promoter	0.000	0.153	T	0.093
rs5030340	10382281	C/T	Intron	0.000	0.073	T	0.050
rs5030351	10385417	C/T	Intron (boundary)	0.000	0.301	T	0.001
rs5030354	10387766	A/C	Intron	-	-	C	0.000
rs5030362	10389562	A/C	Intron	-	-	A	0.000
rs5490	10381827	A/C	5' UTR	0.000	0.250	C	0.000
rs5493	10394794	G/T	Promoter	-	-	T	0.003
rs5495	10395096	A/G	Promoter	0.000	0.006	A	0.001
rs5496	10395447	A/G	Promoter	0.000	0.489	A	0.000
**rs5498**	10395683	A/G	Promoter	0.084	0.494	G	0.429
***IL6* 7p15.3**							
rs11544633	22769164	C/T	Coding exon [L119P]	-	-	C	0.000
rs13306435	22771039	A/T	Coding exon [D162E]	0.000	0.036	A	0.011
**rs1474347**	22768124	G/T	Intron	0.006	0.469	G	0.125
**rs1524107**	22768219	C/T	Intron (boundary)	0.037	0.374	T	0.421
rs1548216	22769773	C/G	Intron	0.000	0.208	C	0.050
**rs1554606**	22768707	G/T	Intron	0.006	0.464	T	0.177
rs2069830	22767137	C/T	Coding exon [P32S]	0.000	0.106	T	0.000
**rs2069832**	22767433	A/G	Intron	0.007	0.473	A	0.119
**rs2069835**	22767871	C/T	Intron	0.000	0.111	C	0.069
rs2069838	22768479	C/T	Intron (boundary)	0.000	0.097	T	0.000
**rs2069840**	22768572	C/G	Intron	0.064	0.318	G	0.142
rs2069842	22769310	A/G	Intron (boundary)	0.000	0.116	A	0.000
rs2069843	22769994	A/G	Intron	0.000	0.144	A	0.049
**rs2069845**	22770149	A/G	Intron	0.000	0.446	G	0.177
rs2069847	22770608	A/G	Intron	-	-	A	0.005

db SNP ID[a]	SNP position	Alleles	Role [amino acid change]	Prior studies min MAF[b]	Prior studies max MAF[b]	Present study minor allele	Present study MAF
rs2069849	22771156	C/T	Coding exon [F201F]	0.000	0.185	T	0.049
rs4335044	22765884	A/T	Promoter	0.000	0.000	T	0.000
***MTHFR* 1p36.22**							
**rs11121832**	11860120	C/T	Intron	0.073	0.351	T	0.237
**rs12121543**	11854671	A/C	Intron (boundary)	0.097	0.337	A	0.361
rs13306560	11866183	A/G	Promoter	0.000	0.051	A	0.065
rs1413355	11862935	A/G	Intron (boundary)	-	-	G	0.000
**rs1476413**	11852300	A/G	Intron (boundary)	0.111	0.440	A	0.444
rs1537516	11847861	C/T	3' UTR	0.043	0.217	T	0.287
rs17037388	11858036	A/G	Intron	-	-	G	0.265
**rs17037390**	11860843	A/G	Intron	0.075	0.223	A	0.273
rs17037404	11863277	A/G	Intron (boundary)	0.000	0.097	A	0.027
rs17367504	11862778	A/G	Intron	0.075	0.175	G	0.253
rs17367629	11865236	C/T	Intron	-	-	T	0.240
**rs17421462**	11856847	A/G	Intron	0.000	0.122	A	0.077
**rs17421511**	11857788	A/G	Intron	0.009	0.234	A	0.156
**rs1801131**	11854476	A/C	Coding exon [E429A]	0.111	0.392	C	0.415
**rs1801133**	11856378	C/T	Coding exon [A222V]	0.080	0.475	T	0.109
**rs1994798**	11854755	C/T	Intron (boundary)	0.189	0.479	C	0.493
rs2066461	11861348	A/C	Coding exon [T115T]	0.006	0.006	A	0.000
rs2066466	11861276	A/G	Coding exon [T139T]	0.005	0.021	A	0.000
**rs2066471**	11860458	A/G	Intron (boundary)	0.000	0.179	A	0.157
rs2077360	11848879	A/G	3' UTR	-	-	A	0.000
**rs2184226**	11847436	A/G	3' UTR	0.000	0.140	G	0.068
rs28484963	11847742	G/T	3' UTR	-	-	G	0.000
**rs3737965**	11866451	C/T	Promoter	0.055	0.093	T	0.165
**rs3753582**	11865542	G/T	Intron	0.065	0.134	G	0.191
**rs3753584**	11864586	A/G	Intron	0.075	0.176	G	0.260
rs3753588	11863904	A/G	Intron	-	-	A	0.228
rs41452445	11858584	A/G	Intron	-	-	G	0.023
rs4845884	11846447	A/G	3' UTR	0.000	0.264	G	0.000
rs4846048	11846252	A/G	3' UTR	0.073	0.482	G	0.032
**rs4846049**	11850365	G/T	3' UTR	0.186	0.482	T	0.474
rs4846050	11852969	C/T	Intron	-	-	T	0.001
**rs4846052**	11857951	C/T	Intron	0.148	0.500	T	0.490
**rs6541003**	11855867	A/G	Intron	0.177	0.500	G	0.499
rs6700268	11851950	C/G	Intron	0.000	0.051	C	0.000
rs7518348	11851118	A/G	Intron	0.000	0.380	A	0.005
**rs7533315**	11860683	C/T	Intron	0.081	0.313	T	0.239
rs7547068	11862331	A/G	Intron	-	-	A	0.000
**rs9651118**	11862214	C/T	Intron	0.000	0.414	C	0.329
***NOS3* 7q36.1**							
rs1008140	150705610	C/T	Intron	-	-	C	0.043
rs1541861	150697333	A/C	Intron	-	-	C	0.261
**rs1800779**	150689943	A/G	Promoter	0.073	0.468	G	0.219
**rs1800783**	150689397	A/T	Promoter	0.058	0.418	A	0.224
**rs1808593**	150708302	G/T	Downstream	0.135	0.240	G	0.183
**rs2853792**	150699877	A/G	Intron	-	-	G	0.310
rs2853795	150703242	A/G	Intron	-	-	G	0.226
**rs3793342**	150695195	C/T	Intron	0.066	0.205	T	0.159
**rs3834873**	150703742/43	I/D[c]	Intron	-	-	I	0.166
rs3918166	150693556	A/G	Coding exon [R112Q]	0.000	0.121	G	0.000
**rs3918169**	150694606	A/G	Intron	-	-	G	0.200
rs3918171	150697095	A/G	Intron	-	-	A	0.047
**rs3918181**	150701783	A/G	Intron	-	-	A	0.304
**rs3918186**	150702432	A/T	Intron	0.069	0.167	T	0.161
**rs3918188**	150702781	A/C	Intron	0.178	0.425	A	0.323
rs3918194	150705200	I/D[c]	Intron	-	-	D	0.000
rs3918198	150706377	A/C	Intron (boundary)	-	-	A	0.000
**rs3918204**	150708459/60	I/D[c]	Intron	-	-	D	0.185
rs3918226	150690176	C/T	Promoter	-	-	T	0.004
**rs3918227**	150700946	A/C	Intron	0.000	0.135	A	0.066
**rs6951150**	150681914	C/T	Promoter	0.000	0.455	T	0.222

db SNP ID[a]	SNP position	Alleles	Role [amino acid change]	Prior studies min MAF[b]	Prior studies max MAF[b]	Present study minor allele	Present study MAF
**rs743506**	150706915	A/G	Intron	0.149	0.476	G	0.190
**rs743507**	150707488	A/G	Intron	0.139	0.403	G	0.189
rs7830	150709571	A/C	3' UTR	0.122	0.445	A	0.436
**rs891512**	150708089	A/G	Downstream	0.000	0.296	A	0.140
***PNP* 14q11.2**							
**rs1049562**	20940515	A/G	Coding exon [H20H]	0.106	0.265	A	0.137
**rs1049564**	20940606	C/T	Coding exon [G51S]	0.121	0.350	T	0.139
rs12101049	20939701	A/G	Intron	-	-	A	0.000
**rs1617940**	20941413	A/T	Intron	0.241	0.346	T	0.161
rs17112168	20936978	A/C	Promoter	-	-	A	0.008
**rs1713420**	20942744	C/T	Intron (boundary)	0.093	0.362	C	0.139
**rs1713421**	20942451	A/C	Intron	0.094	0.362	C	0.142
**rs1756369**	20943504	A/T	Intron (boundary)	0.139	0.381	T	0.159
rs1760933	20945488	A/G	3' UTR	-	-	A	0.044
**rs1760935**	20941613	C/T	Intron	0.122	0.457	C	0.137
**rs1760940**	20938251	G/T	Intron	0.102	0.233	G	0.141
rs17878900	20945355	C/T	3' UTR	-	-	T	0.035
rs17879107	20940360	G/T	Intron	-	-	G	0.018
rs17880290	20937412	A/G	Promoter	-	-	A	0.029
rs17880503	20935743	C/T	Promoter	-	-	C	0.013
rs17881184	20943565	A/G	Intron	-	-	G	0.000
rs17881554	20943281	A/T	Coding exon [A174A]	-	-	A	0.000
**rs17882804**	20941303/04	I/D[c]	Intron	-	-	I	0.158
rs17882836	20939039	A/G	Intron	-	-	A	0.008
rs17883795	20945571	A/G	3' UTR	-	-	A	0.020
rs17885714	20941715	A/G	Intron	-	-	G	0.023
rs17885781	20938710/11	I/D[c]	Intron	-	-	D	0.000
rs17885917	20940735	A/G	Intron (boundary)	-	-	G	0.012
rs17886095	20940798	A/G	Intron	-	-	A	0.019
rs3790062	20937111	A/G	Promoter	0.000	0.037	A	0.008
rs3790064	20941069	A/G	Intron	0.014	0.134	G	0.038
***S1PR1* 1p21.2**							
**rs1411017**	101703249	A/G	Intron	0.045	0.417	G	0.090
**rs17100954**	101704029	A/G	Intron	0.065	0.067	A	0.091
**rs3737577**	101704532	A/C	5' UTR	0.018	0.306	A	0.096
**rs3753194**	101702825	C/T	Intron	0.012	0.371	C	0.071
rs4987248	101702546	C/G	5' UTR	0.046	0.244	C	0.077
rs7549921	101705535	C/G	Coding exon [P332R]	0.000	0.000	G	0.000
***SOD2* 6q25.3**							
rs10370	160101532	A/C	Intron	-	-	C	0.321
rs12195992	160109524	C/G	Intron	-	-	G	0.000
rs12526686	160112013	C/T	Intron	-	-	T	0.000
rs1800665	160107091	A/G	Intron	-	-	A	0.002
**rs1800666**	160110140	A/G	Intron	-	-	A	0.264
**rs2758331**	160105070	A/C	Intron	0.116	0.461	C	0.482
**rs2758332**	160106088	A/C	Intron (boundary)	-	-	C	0.469
**rs2758334**	160110454	C/T	Intron	-	-	T	0.482
rs2758340	160112709	A/C	Intron	-	-	A	0.000
rs2758345	160114793	C/G	Promoter	-	-	G	0.001
**rs2842960**	160113321	C/T	Intron	0.116	0.474	C	0.465
rs3798215	160104838	A/G	Intron	-	-	G	0.241
**rs5746088**	160114726	A/G	Promoter	-	-	A	0.052
rs5746099	160113599	A/C	Intron (boundary)	-	-	C	0.000
rs5746104	160112855	C/G	Intron	-	-	G	0.048
**rs5746105**	160112638	C/T	Intron	0.181	0.420	C	0.321
rs5746108	160112560	A/G	Intron	-	-	A	0.000
rs5746109	160112496	A/G	Intron	-	-	G	0.007
rs5746110	160112387	A/G	Intron	-	-	G	0.000
rs5746111	160109611	C/G	Intron	-	-	C	0.005
rs5746114	160108721	A/G	Intron	-	-	G	0.000
rs5746123	160106703	A/G	Intron	-	-	A	0.000
**rs5746136**	160103084	A/G	Intron	0.152	0.421	A	0.306
rs5746141	160102710	A/G	Intron	0.000	0.086	A	0.038

db SNP ID[a]	SNP position	Alleles	Role [amino acid change]	Prior studies min MAF[b]	Prior studies max MAF[b]	Present study minor allele	Present study MAF
**rs6912979**	160120799	C/T	Promoter	0.200	0.421	C	0.291
**rs8031**	160100640	A/T	Intron	0.116	0.461	T	0.485
rs9457709	160111917	A/G	Intron	-	-	A	0.000
***TNF* 6p21.33**							
rs17207127	31541948	C/T	3' UTR	-	-	T	0.007
rs17207134	31542113	C/T	Downstream	-	-	C	0.000
**rs1799964**	31542308	C/T	Downstream	-	-	C	0.358
rs1800610	31543827	C/T	Intron	-	-	T	0.074
**rs1800630**	31542476	A/C	Downstream	-	-	A	0.268
rs2228088	31543605	G/T	Coding exon [R29R]	0.000	0.065	T	0.000
rs3093544	31541779	A/G	3' UTR	-	-	G	0.000
rs3093547	31541848	A/T	3' UTR	-	-	A	0.044
**rs3093661**	31543758	A/G	Intron	0.003	0.163	A	0.093
**rs3093662**	31544189	A/G	Intron	0.018	0.189	G	0.104
**rs3093664**	31544642	A/G	Intron	-	-	G	0.106
rs3093665	31545391	A/C	3' UTR	0.004	0.061	C	0.010
rs3179060	31543672	A/C	Coding exon [H52N]	-	-	A	0.000
rs4248159	31542580	A/C	Downstream	-	-	A	0.001
rs4645843	31544562	C/T	Coding exon [P84L]	0.000	0.010	T	0.000
***VCAM1* 1p21.2**							
rs1041163	101183825	C/T	Promoter	0.071	0.186	C	0.177
**rs1409419**	101183396	C/T	Promoter	0.355	0.411	T	0.401
**rs2209627**	101199147	A/G	Intron	0.011	0.116	G	0.072
**rs2392221**	101190173	C/T	Intron (boundary)	-	-	T	0.173
rs3170794	101184584	C/T	Promoter	0.000	0.153	C	0.003
rs3176859	101186870	G/T	Intron	-	-	T	0.035
**rs3176860**	101187219	A/G	Intron	0.305	0.500	A	0.433
**rs3176861**	101187321	C/T	Intron	0.010	0.256	T	0.399
rs3176862	101187972	C/G	Intron	0.000	0.164	G	0.003
rs3176863	101188192	A/G	Intron	0.037	0.337	A	0.182
**rs3176867**	101194205	C/T	Intron	0.005	0.372	T	0.287
**rs3176870**	101197183	A/G	Intron	0.009	0.250	A	0.087
**rs3176871**	101197289	A/G	Intron	0.012	0.093	A	0.051
**rs3176874**	101199886	A/G	Intron	0.036	0.281	G	0.184
rs3176876	101200608	A/G	Intron	0.250	0.436	G	0.384
**rs3176877**	101203395	A/T	Intron	0.278	0.500	A	0.450
**rs3176878**	101203698	C/T	Coding exon [D601D]	0.000	0.234	T	0.071
rs3181087	101184175	A/T	Promoter	0.000	0.053	T	0.000
**rs3181088**	101198708	C/T	Intron	0.000	0.191	T	0.138
rs3181089	101198826	C/T	Intron	0.000	0.115	T	0.000
**rs3181092**	101204644	A/G	Downstream	0.241	0.500	A	0.435
**rs3765685**	101192993	A/G	Intron	0.071	0.149	G	0.168
rs3783597	101183724	C/G	Promoter	0.000	0.019	G	0.000
rs3783599	101183887	C/T	Promoter	0.000	0.073	T	0.000
rs3783601	101184061	A/G	Promoter	0.000	0.027	G	0.000
rs3783603	101184269	A/G	Promoter	0.000	0.019	A	0.000
rs3783606	101185034	G/T	Promoter	0.000	0.000	G	0.000
rs3783609	101185363	A/G	5' UTR	0.000	0.116	A	0.000
rs3783613	101196787	C/G	Coding exon [G321A]	0.000	0.173	C	0.000
rs3783617	101203881	C/G	3' UTR	-	-	C	0.000
rs3783624	101204798	A/G	Downstream	0.000	0.036	G	0.000
rs3917009	101189410	C/T	Intron	0.030	0.125	T	0.022
**rs3917010**	101190866	A/C	Intron	0.000	0.250	C	0.425
rs3917014	101197384	A/G	Intron	0.009	0.244	A	0.452
**rs3917016**	101201147	A/T	Intron	0.036	0.227	A	0.180
**rs3917018**	101202222	A/G	Intron	0.245	0.500	A	0.443
**rs3917019**	101202354	A/G	Intron	0.259	0.417	A	0.289
**rs3917022**	101204883	C/T	Downstream	0.027	0.148	C	0.175
rs3917026	101184824	C/T	Promoter	0.000	0.033	C	0.000
rs3917032	101187123	A/C	Intron	-	-	A	0.000
rs3917033	101187613	C/T	Intron	-	-	C	0.000
rs3917057	101196175	A/G	Intron	0.000	0.048	G	0.000

[a]SNPs that were included in the final analysis (*n* = 170) are bolded. The remaining SNPs were excluded because of poor genotyping efficiency (< 95%), monomorphic genotype data, deviation from Hardy-Weinberg equilibrium (< 0.0001), or low MAF (< 5%). [b]http://snpper.chip.org/bio/snpper-enter/. SNPper is a web-based tool to retrieve known SNPs from public databases. MAF data are not available in SNPper for some of the known SNPs. [c]Insertion/deletion polymorphism.

Table S2. Characteristics of the subcohort in the present study (*n* = 1,375) and participants in the overall cohort study (*n* = 20,033) [*n* (%) or mean ± SD].

**Characteristic**	**Subcohort**[a]	**Overall cohort participants**
Sex		
Women	800 (58.2)	11870 (59.3)
Men	575 (41.8)	8163 (40.8)
Age (years)	38.6 ± 9.7	36.9 ± 10.4
Body mass index (kg/m^2)	19.9 ± 3.3	19.8 ± 3.2
Education (years)	3.1 ± 3.7	3.5 ± 3.8
Smoking status		
Never	877 (63.8)	13510 (67.5)
Past	90 (6.6)	1255 (6.3)
Current	408 (29.7)	5260 (26.3)
Systolic blood pressure (mmHg)	116.5 ± 17.3	116.6 ± 17.2
Diastolic blood pressure (mmHg)	75.0 ± 10.9	74.9 ± 11.2
Well arsenic (µg/L)	80.8 ± 101.3	81.7 ± 105.9
Urinary arsenic (µg/L)	114.9 ± 131.8	118.7 ± 144.5
Urinary creatinine (mg/dL)	54.0 ± 42.7	53.8 ± 42.6
Urinary arsenic (µg/g creatinine)	257.6 ± 322.0	259.2 ± 285.3

[a]The subcohort included 56 CVD cases.

Table S3. Association between baseline well-water arsenic and CVD, CHD, and stroke.

Well-water arsenic (μg/L)	Mean[a]	Subcohort[b] (*n*)	CVD cases (*n*)	CVD aHR (95% CI)[c]	CHD cases (*n*)	CHD aHR (95% CI)[c]	Stroke cases (*n*)	Stroke aHR (95% CI)[c]
0.1-16	4.3	453	125	1.00	69	1.00	44	1.00
17-85	46.7	452	147	1.24 (0.84, 1.84)	86	1.30 (0.83, 2.01)	50	1.14 (0.65, 1.98)
86-864	191.2	454	174	1.69 (1.15, 2.50)	82	1.40 (0.88, 2.23)	71	1.87 (1.06, 3.29)
Per SD (101.3 μg/L)		1359	446	1.21 (1.08, 1.37)	237	1.17 (1.01, 1.35)	165	1.19 (1.02, 1.40)

[a]Category-specific mean values of well-water arsenic in the subcohort. [b]Data on well-water arsenic were missing on 16 subjects in the subcohort. [c]Adjusted for sex, age, BMI, smoking status (never, past, and current), educational attainment, systolic blood pressure, diabetes status, and change in creatinine-adjusted urinary arsenic between visits.

Table S4. Nominally significant interactions between well-water arsenic and SNPs in CHD and stroke.

db SNP ID	Genotype	MAF (%)	aHR (95%CI) well-water arsenic[a]	aHR (95%CI) SNP[a]	aHR (95%CI) joint[a]	P^b	$P_{adj}{}^c$
**CHD**							
AS3MT							
rs1046778	TC+CC vs. TT	C (34.8)	0.96 (0.74, 1.24)	0.96 (0.63, 1.46)	1.25 (0.84, 1.85)	0.043	0.315
rs11191454	AG+GG vs. AA	G (17.2)	1.06 (0.88, 1.26)	0.91 (0.55, 1.52)	1.33 (0.88, 2.01)	0.036	0.290
rs12573221	AC+CC vs. AA	C (12.1)	1.05 (0.87, 1.27)	0.72 (0.43, 1.22)	1.17 (0.76, 1.78)	0.007	0.257
CBS							
rs1005585	AG+GG vs. AA	G (7.8)	1.10 (0.93, 1.29)	0.48 (0.24, 0.96)	0.93 (0.55, 1.58)	0.009	0.257
rs11700748	TC+TT vs. CC	T (38.9)	0.86 (0.62, 1.19)	0.79 (0.50, 1.23)	1.04 (0.68, 1.58)	0.018	0.257
rs2124459	TC+CC vs. TT	C (41.1)	0.91 (0.65, 1.27)	0.83 (0.50, 1.35)	1.08 (0.67, 1.73)	0.050	0.321
rs2849727	TC+TT vs. CC	T (33.9)	0.92 (0.69, 1.24)	0.84 (0.55, 1.29)	1.08 (0.72, 1.61)	0.050	0.321
rs3788050	GT+TT vs. GG	T (8.2)	1.09 (0.93, 1.29)	0.52 (0.27, 1.01)	0.96 (0.58, 1.58)	0.018	0.257
rs706208	TC+CC vs. TT	C (38.5)	0.88 (0.63, 1.24)	0.77 (0.49, 1.21)	1.02 (0.67, 1.56)	0.031	0.275
GSTO1							
rs1147611	CA+AA vs. CC	A (30.2)	0.98 (0.77, 1.25)	1.25 (0.80, 1.95)	1.72 (1.13, 2.60)	0.028	0.275
rs11509438	GA+AA vs. GG	A (10.1)	1.11 (0.94, 1.32)	1.23 (0.72, 2.09)	2.07 (1.36, 3.15)	0.028	0.275
rs2282326	AC+CC vs. AA	C (30.0)	1.00 (0.80, 1.26)	1.27 (0.82, 1.97)	1.74 (1.15, 2.63)	0.036	0.290
ICAM1							
rs281432	GG vs. CG+CC	C (49.5)	1.06 (0.89, 1.27)	0.95 (0.59, 1.55)	1.56 (1.04, 2.34)	0.007	0.257
IL6							
rs2069835	TC+CC vs. TT	C (6.9)	1.10 (0.94, 1.30)	0.74 (0.40, 1.39)	1.27 (0.77, 2.12)	0.031	0.275
MTHFR							
rs12121543	CA+AA vs. CC	A (36.1)	0.92 (0.68, 1.24)	0.65 (0.41, 1.02)	0.92 (0.61, 1.39)	0.016	0.257
rs17421462	GG vs. GA+AA	A (7.7)	0.75 (0.49, 1.15)	0.64 (0.37, 1.11)	0.80 (0.47, 1.36)	0.029	0.275
rs1801131	AC+CC vs. AA	C (41.5)	0.84 (0.58, 1.22)	0.66 (0.41, 1.06)	0.92 (0.59, 1.43)	0.015	0.257
NOS3							
rs1800783	TA+AA vs. TT	A (22.4)	0.98 (0.77, 1.23)	0.67 (0.43, 1.05)	0.94 (0.63, 1.39)	0.015	0.257
rs6951150	TC+TT vs. CC	T (22.2)	0.97 (0.77, 1.24)	0.68 (0.44, 1.07)	0.96 (0.65, 1.44)	0.014	0.257
SOD2							
rs2758334	TC+CC vs. TT	T (48.2)	0.87 (0.61, 1.26)	0.72 (0.45, 1.17)	0.96 (0.61, 1.51)	0.042	0.315
VCAM1							
rs1409419	CC vs. TC+TT	T (40.1)	1.07 (0.86, 1.32)	0.54 (0.33, 0.88)	0.83 (0.56, 1.24)	0.027	0.275
rs2209627	AA vs. AG+GG	G (7.2)	0.61 (0.37, 1.01)	0.57 (0.32, 1.00)	0.70 (0.40, 1.23)	0.008	0.257

db SNP ID	Genotype	MAF (%)	aHR (95%CI) well-water arsenic[a]	aHR (95%CI) SNP[a]	aHR (95%CI) joint[a]	P^b	P_{adj}^c
rs3176867	CC vs. TC+TT	T (28.7)	1.03 (0.82, 1.29)	1.05 (0.68, 1.62)	1.51 (1.01, 2.26)	0.027	0.275
rs3176871	GG vs. GA+AA	A (5.1)	0.76 (0.53, 1.08)	0.39 (0.21, 0.71)	0.48 (0.27, 0.88)	0.011	0.257
rs3176878	CC vs. TC+TT	T (7.1)	0.65 (0.40, 1.07)	0.63 (0.36, 1.11)	0.78 (0.45, 1.35)	0.014	0.257
rs3917014	GG vs. AG+AA	A (45.2)	1.12 (0.96, 1.31)	0.75 (0.36, 1.56)	1.55 (0.87, 2.73)	0.003	0.260
**Stroke**							
AS3MT							
rs10786719	AG+GG vs. AA	G (43.8)	0.82 (0.52, 1.28)	0.88 (0.48, 1.61)	1.16 (0.64, 2.08)	0.050	0.440
rs11191439	TT vs. TC+CC	C (5.4)	0.70 (0.41, 1.20)	0.66 (0.30, 1.44)	0.82 (0.38, 1.76)	0.044	0.440
CBS							
rs8132811	CT+TT vs. CC	T (13.0)	1.14 (0.95, 1.36)	0.55 (0.29, 1.04)	0.97 (0.57, 1.64)	0.021	0.440
GSTO1							
rs1147611	CA+AA vs. CC	A (30.2)	1.00 (0.77, 1.29)	1.15 (0.67, 1.98)	1.64 (0.99, 2.73)	0.027	0.440
ICAM1							
rs281432	GG vs. CG+CC	C (49.5)	1.08 (0.90, 1.31)	0.92 (0.52, 1.61)	1.85 (1.14, 3.01)	8.3×10^{-5}	0.014
NOS3							
rs1800779	AG+GG vs. AA	G (21.9)	1.03 (0.82, 1.28)	0.96 (0.57, 1.61)	1.36 (0.84, 2.20)	0.034	0.440
rs1800783	TA+AA vs. TT	A (22.4)	0.99 (0.79, 1.24)	0.76 (0.45, 1.27)	1.10 (0.69, 1.76)	0.013	0.440
rs3793342	CT+TT vs. CC	T (15.9)	1.02 (0.82, 1.26)	0.89 (0.52, 1.55)	1.29 (0.79, 2.12)	0.023	0.440
rs3918169	AG+GG vs. AA	G (20.0)	1.03 (0.83, 1.28)	0.82 (0.48, 1.40)	1.18 (0.73, 1.92)	0.031	0.440
rs6951150	CT+TT vs. CC	T (22.2)	0.93 (0.74, 1.17)	0.67 (0.40, 1.14)	1.01 (0.63, 1.62)	0.002	0.193
VCAM1							
rs3176867	CC vs. TC+TT	T (28.7)	1.00 (0.78, 1.28)	0.78 (0.47, 1.30)	1.23 (0.76, 1.99)	0.007	0.377

[a]Adjusted HR in association with a 1-SD increase in well-water arsenic (101.3 µg/L) and "at-risk" genotype (s) of SNPs, and joint effect between well-water arsenic and SNPs, adjusting for sex, age, BMI, smoking status (never, past, and current), educational attainment, systolic blood pressure, diabetes status, and change in creatinine-adjusted urinary arsenic between visits. [b]Nominal *P* values from *1df* tests for multiplicative interactions between a 1-SD increase well-water arsenic and SNPs. [c]FDR adjusted *P* values.

Table S5. Nominally significant interactions between urinary creatinine-adjusted arsenic and SNPs in CVD, CHD, and stroke.

db SNP ID	Genotype	MAF (%)	aHR (95%CI) urinary arsenic[a]	aHR (95%CI) SNP[a]	aHR (95%CI) joint[a]	*P*[b]
**CVD**						
AS3MT						
rs12573221	AC+CC vs. AA	C (12.1)	0.95 (0.71, 1.27)	0.64 (0.39, 1.05)	0.99 (0.64, 1.54)	0.011
rs4290163	GT+TT vs. GG	T (42.2)	0.81 (0.56, 1.18)	0.78 (0.50, 1.23)	1.11 (0.72, 1.70)	0.011
CBS						
rs1005585	AG+GG vs. AA	G (7.8)	1.01 (0.75, 1.35)	0.44 (0.25, 0.76)	1.02 (0.63, 1.64)	4.3×10^{-7}
rs3788050	GT+TT vs. GG	T (8.2)	1.00 (0.75, 1.33)	0.49 (0.29, 0.82)	1.09 (0.69, 1.73)	1.1×10^{-6}
rs8132811	CT+TT vs. CC	T (13.0)	1.00 (0.74, 1.34)	0.55 (0.35, 0.89)	1.00 (0.65, 1.54)	0.002
ICAM1						
rs281432	GG vs. CG+CC	C (49.5)	1.01 (0.75, 1.35)	1.16 (0.79, 1.72)	1.68 (1.12, 2.52)	0.014
SOD2						
rs2758331	CA+AA vs. CC	C (48.2)	0.77 (0.51, 1.15)	0.69 (0.44, 1.08)	0.97 (0.63, 1.50)	0.010
rs2758334	TC+CC vs. TT	T (48.2)	0.72 (0.49, 1.07)	0.61 (0.39, 0.95)	0.86 (0.56, 1.31)	0.005
rs8031	TA+AA vs. TT	T (48.5)	0.73 (0.49, 1.10)	0.66 (0.42, 1.02)	0.90 (0.59, 1.38)	0.008
**CHD**						
AS3MT						
rs12573221	AC+CC vs. AA	C (12.1)	0.95 (0.69, 1.30)	0.70 (0.41, 1.18)	1.03 (0.63, 1.69)	0.035
CBS						
rs1005585	AG+GG vs. AA	G (7.8)	1.01 (0.74, 1.37)	0.42 (0.21, 0.83)	1.01 (0.57, 1.79)	0.0003
rs3788050	GT+TT vs. GG	T (8.2)	0.99 (0.73, 1.35)	0.45 (0.23, 0.87)	1.02 (0.59, 1.79)	0.0009
VCAM1						
rs1409419	CC vs. TC+TT	T (40.1)	0.96 (0.69, 1.34)	0.47 (0.28, 0.78)	0.84 (0.54, 1.31)	0.002
rs2209627	AA vs. AG+GG	G (7.2)	0.45 (0.19, 1.08)	0.49 (0.25, 0.99)	0.57 (0.29, 1.14)	0.043
rs3176878	CC vs. TC+TT	T (7.1)	0.45 (0.18, 1.16)	0.52 (0.25, 1.05)	0.62 (0.31, 1.25)	0.050
**Stroke**						
CBS						
rs8132811	CT+TT vs. CC	T (13.0)	0.92 (0.63, 1.33)	0.44 (0.22, 0.90)	0.89 (0.49, 1.63)	0.003
ICAM1						
rs281432	GG vs. CG+CC	C (49.5)	0.96 (0.67, 1.39)	0.97 (0.55, 1.72)	1.60 (0.87, 2.93)	0.005

[a]Adjusted HR in association with a 1-SD increase in urinary arsenic (322 µg/g creatinine) and "at-risk" genotype (s) of SNPs, and joint effect between well-water arsenic and SNPs, adjusting for sex, age, BMI, smoking status (never, past, and current), educational attainment, systolic blood pressure, diabetes status, and change in creatinine-adjusted urinary arsenic between visits. [b]Nominal *P* values from *1df* tests for multiplicative interactions between a 1-SD increase urinary arsenic and SNPs.

Table S6. Nominally significant associations between SNPs and CVD, CHD, and stroke.

db SNP ID	Genotype	MAF (%)	aHR (95% CI)[a]	P^b	$P_{adj}{}^c$
**CVD**					
AS3MT					
rs10748835	AG+GG vs. AA	A (43.9)	1.44 (1.04, 1.99)	0.027	0.417
rs3740393	CG+CC vs. GG	C (18.9)	1.47 (1.09, 1.97)	0.011	0.315
CBS					
rs1789953	CT+TT vs. CC	T (23.8)	0.69 (0.51, 0.94)	0.018	0.384
rs234706	GA+AA vs. GG	A (20.9)	1.34 (1.01, 1.79)	0.043	0.421
CYBA					
rs3794624	GA+AA vs. GG	A (10.7)	1.43 (1.05, 1.95)	0.023	0.403
HMOX1					
rs2285112	GA+AA vs. GG	A (46.6)	0.73 (0.54, 0.99)	0.042	0.421
ICAM1					
rs281432	GC+CC vs. GG	C (49.5)	0.65 (0.48, 0.87)	0.004	0.176
IL6					
rs2069832	GA+AA vs. GG	A (11.9)	0.60 (0.40, 0.90)	0.013	0.319
MTHFR					
rs1801133	CT+TT vs. CC	T (10.9)	1.58 (1.16, 2.17)	0.004	0.176
NOS3					
rs2853792	AG+GG vs. AA	G (31.0)	0.51 (0.38, 0.69)	1.0×10^{-5}	0.002
SOD2					
rs5746088	GA+AA vs. GG	A (5.2)	0.30 (0.17, 0.51)	1.0×10^{-5}	0.001
rs5746136	GA+AA vs. GG	A (30.6)	1.35 (1.01, 1.79)	0.044	0.421
VCAM1					
rs2392221	CT+TT vs. CC	T (17.3)	0.63 (0.45, 0.89)	0.008	0.281
rs3176860	GA+AA vs.GG	A (43.3)	1.39 (1.01, 1.92)	0.044	0.421
rs3176870	GA+AA vs. GG	A (8.7)	1.49 (1.05, 2.11)	0.024	0.403
rs3176871	GA+AA vs. GG	A (5.1)	1.57 (1.01, 2.44)	0.044	0.421
**CHD**					
AS3MT					
rs3740393	CG+CC vs. GG	C (18.9)	1.46 (1.03, 2.08)	0.035	0.542
CBS					
rs1789953	CT+TT vs. CC	T (23.8)	0.65 (0.45, 0.94)	0.021	0.457
rs234705	CT+TT vs. CC	T (21.9)	1.49 (1.07, 2.10)	0.020	0.457
rs234706	GA+AA vs. GG	A (20.9)	1.50 (1.07, 2.11)	0.020	0.457
CYBA					
rs3794624	GA+AA vs. GG	A (10.7)	1.63 (1.11, 2.40)	0.014	0.457
GSTO1					
rs1147611	CA+AA vs. CC	A (30.2)	1.47 (1.05, 2.07)	0.027	0.457
rs11509438	GA+AA vs. GG	A (10.1)	1.54 (1.05, 2.26)	0.027	0.457
rs2282326	AC+CC vs. AA	C (30.0)	1.48 (1.05, 2.09)	0.025	0.457
NOS3					
rs2853792	AG+GG vs. AA	G (31.0)	0.49 (0.34, 0.70)	0.0001	**0.018**
SOD2					
rs5746088	GA+AA vs. GG	A (5.2)	0.29 (0.15, 0.58)	0.0004	**0.037**
VCAM1					
rs2392221	CT+TT vs. CC	T (17.3)	0.57 (0.38, 0.85)	0.006	0.312

db SNP ID	Genotype	MAF (%)	aHR (95% CI)[a]	P^b	P_{adj}^c
**Stroke**					
AS3MT					
rs3740393	CG+CC vs. GG	C (18.9)	1.55 (1.01, 2.37)	0.044	0.690
IL6					
rs2069832	GA+AA vs. GG	A (11.9)	0.53 (0.31, 0.92)	0.025	0.690
MTHFR					
rs17037390	GA+AA vs. GG	A (27.3)	0.63 (0.40, 0.98)	0.039	0.690
rs1801133	CT+TT vs. CC	T (10.9)	2.33 (1.51, 3.61)	0.0001	**0.024**
rs3737965	CT+TT vs. CC	T (16.5)	0.47 (0.28, 0.80)	0.006	0.475
rs3753584	AG+GG vs. AA	G (26.0)	0.61 (0.40, 0.96)	0.031	0.690
NOS3					
rs2853792	AG+GG vs. AA	G (31.0)	0.63 (0.41, 0.97)	0.036	0.690
SOD2					
rs5746088	GA+AA vs. GG	A (5.2)	0.38 (0.17, 0.84)	0.017	0.690
VCAM1					
rs3176870	GA+AA vs. GG	A (8.7)	1.81 (1.10, 2.99)	0.020	0.690
rs3917022	TC+CC vs. TT	C (17.5)	1.54 (1.00, 2.35)	0.049	0.690

[a]Adjusted HR in association with "at-risk" genotype (s) of SNPs, adjusting for sex, age, BMI, smoking status (never, past, and current), educational attainment, systolic blood pressure, and diabetes status. [b]Nominal *P* values from *1df* tests.

[c]FDR adjusted *P* values.

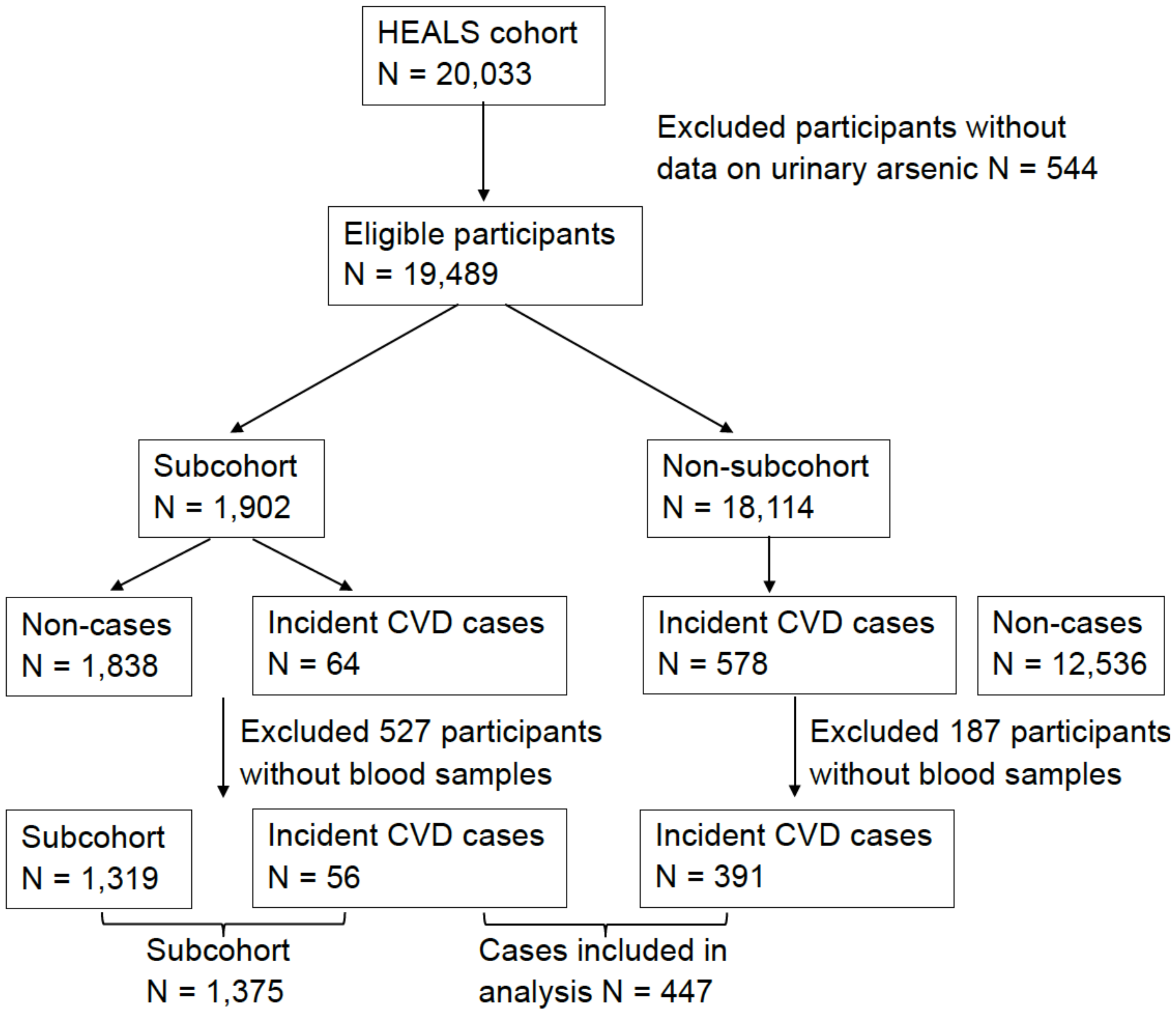

Figure S1. Flow chart of selection of incident CVD cases and the subcohort. Incident CVD cases include fatal and non-fatal CHD, stroke, and other CVD.

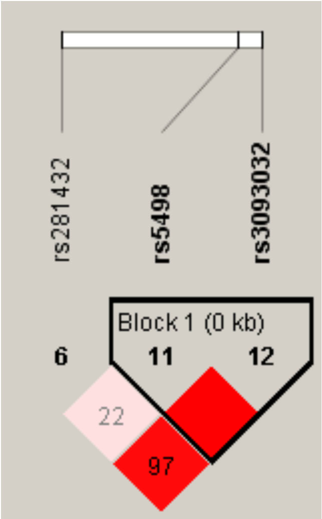

Figure S2. Linkage disequilibrium (LD) plot for *ICAM1* SNPs in Bangladeshi. The LD relationship between each pair of SNPs is indicated by the D prime, derived from the genotypes in the subcohort using the Haploview software. The shading indicates the extent of LD and a greater LD is represented by darker shading.

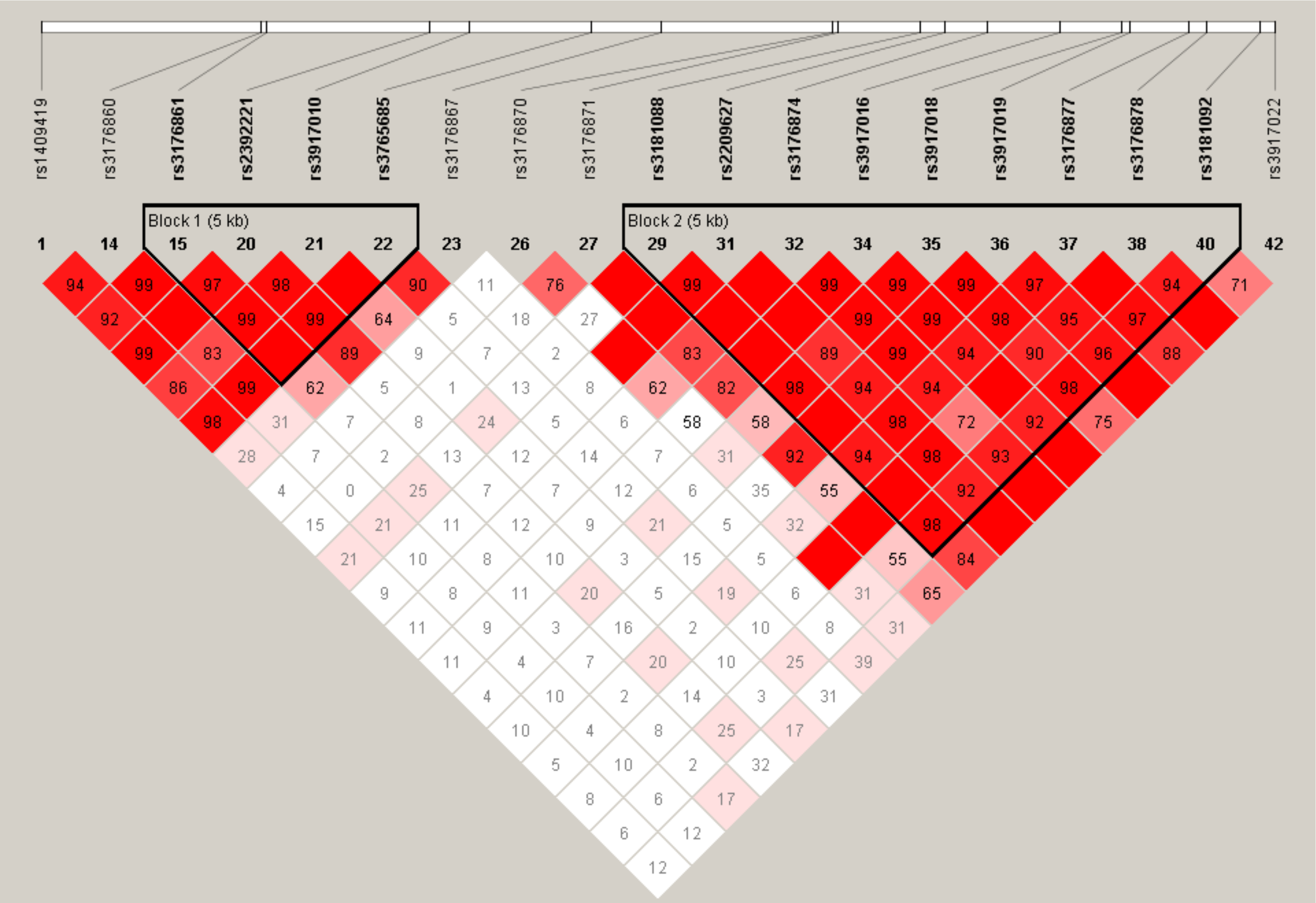
rs1409419
rs3176860
rs3176861
rs2392221
rs3917010
rs3765685
rs3176867
rs3176870
rs3176871
rs3181088
rs2209627
rs3176874
rs3917016
rs3917018
rs3917019
rs3176877
rs3176878
rs3181092
rs3917022
Block 1 (5 kb)
Block 2 (5 kb)
1 14 15 20 21 22 23 26 27 29 31 32 34 35 36 37 38 40 42

Figure S3. Linkage disequilibrium (LD) plot for *VCAM1* SNPs in Bangladeshi. The LD relationship between each pair of SNPs is indicated by the D prime, derived from the genotypes in the subcohort using the Haploview software. The shading indicates the extent of LD and a greater LD is represented by darker shading.
